# Association between Serum Total Bilirubin Level and Patients with Primary Open-Angle Glaucoma in China: A Cross-Sectional, Case-Control Study

**DOI:** 10.1155/2023/8206298

**Published:** 2023-01-19

**Authors:** Mingxi Shao, Shiyun Wang, Yani Wan, Zhenzhen Liu, Yi Ma, Wenjun Cao, Shengjie Li

**Affiliations:** Department of Clinical Laboratory, Eye and ENT Hospital, Shanghai Medical College, Fudan University, Shanghai 200031, China

## Abstract

**Objective:**

To investigate the relationship between peripheral blood total bilirubin (TBIL) levels and the risk of primary open-angle glaucoma (POAG).

**Methods:**

This study was a cross-sectional, case-control study design. Between April 2021 and January 2022, 198 POAG patients and 205 healthy subjects were recruited from the EENT Hospital of Fudan University. Their clinical information (intraocular pressure, central corneal thickness, vertical cup-disk ratios (VCDR), and axial length) and demographic data were collected. Serum levels of TBIL were measured in enzymes using a Roche C702 biochemical analyzer. The POAG subgroups were classified by gender and VCDR: mild (VCDR ≤ 0.64), moderate (VCDR ≤ 0.85), and severe (VCDR > 0.85). Univariate and multivariate logistic regression analyses were performed.

**Results:**

The level of TBIL (11.58 ± 5.16 *μ*mol/L) in the POAG group was higher than that in the control group (10.18 ± 3.38 *μ*mol/L; *p* < 0.05). In the male subgroup, TBIL was also significantly higher than in the normal control group; TBIL levels were lower in the mild subgroup (10.82 ± 4.48 *μ*mol/L), followed by the moderate subgroup (12.00 ± 5.55 *μ*mol/L) and the severe subgroup (14.47 ± 5.45 *μ*mol/L). The results of the multivariate logistic regression analysis showed that high TBIL levels were a risk factor for male POAG, at 1.126 (95% CI 1.009–1.256). Pearson's analysis revealed that TBIL was positively correlated with intraocular pressure (*r* = 0.134, *p* = 0.012), VCDR (*r* = 0.142, *p* = 0.046), anterior chamber depth (*r* = 0.190, *p* = 0.014), and axial length (*r* = 0.179, *p* = 0.019) in the patients. However, no statistical difference (*p* < 0.05) was observed in the female patients with POAG.

**Conclusion:**

The results showed that high levels of TBIL may be related to the pathogenesis of POAG and that the severity of the disease is positively correlated, especially in male patients.

## 1. Introduction

The genetic variability of eye illness results in the common multi-factor-induced optic nerve damage and vision impairment known as glaucoma. The most common cause of permanent blindness is glaucoma, which is characterized by a gradual loss of retinal ganglion cells (RGC) [1]. Primary glaucoma is currently separated into primary open-angle glaucoma (POAG) and primary angle closure glaucoma (PACG), among other classifications, based on the anterior chamber angle structure, etiology, age at start, and intraocular pressure. Clinical manifestations of POAG are usually bilateral, with anterior chamber angle opening, progressive death, and atrophy of RGCs and their associated axons resulting in visual field defects and even blindness [2]. POAG is a group of complex diseases caused by a variety of factors. According to recent studies, high intraocular pressure, genetic variation, vascular damage, oxidative stress, ametropia, and diabetes are all risk factors [3–6].

As an endogenous antioxidant, total bilirubin (TBIL) has been shown to have potential antioxidant activity [7] and to be inversely associated with oxidative stress [8]. However, toxicological studies have shown that a high concentration of bilirubin in serum can result in the deposition of bilirubin in various tissues, causing diseases such as brain injury, cerebral palsy, and mental disorders [9]. Hyperbilirubinemia has been associated with the World Health Organization's severe functional classification, higher right atrial pressure, higher brain natriuretic peptide levels, and larger indolent right ventricular Doppler signals [10, 11].

Based on the increasing amount of evidence of increased oxidative and cytotoxic stress in glaucomatous tissues [12–14], oxidatively modified proteins or oxidative stress-related products may be particularly valuable as glaucoma-related biomarkers. Thus, we hypothesized that bilirubin, an oxidative and cytotoxic stress biomarker, plays a vital role in the physiological and pathological conditions of glaucoma. However, only a few previous researches have been conducted on the association between bilirubin levels and POAG, and they have been limited by their sample sizes [15]. To investigate the connection between bilirubin and POAG, we did a large sample, cross-sectional, case-control investigation in the current study.

## 2. Materials and Methods

### 2.1. Subjects

The Eye and ENT Hospital's Ethics Review Committee at Shanghai's Fudan University granted the study approval. The design of this study adheres to the tenets of the Declaration of Helsinki. All patients gave their informed permission. Patients with POAG were sequentially enrolled from Fudan University's Department of Ophthalmology, Visual Science of the Eye, and ENT Hospital between April 2021 and January 2022. Control subjects were chosen among those who had annually received health checks during the research period.

### 2.2. Examination

All POAG patients enrolled in this study underwent medical examinations and kidney function assessments. Liver function, heart rate, infectious diseases, blood pressure, and X-rays were included. Additionally, all POAG patient received a routine eye examination by glaucoma specialists. Refractive state, fundus, IOP, axial length, anterior chamber depth, central corneal thickness, and visual field were all assessed throughout this process. Additionally, anterior chamber angle arthroscopy and slit-lamp biomicroscopy were performed. Using an automated Octopus perimeter (Haag-Streit AG, Switzerland), as previously reported [13], mean defect (MD) and mean sensitivity (MS) were calculated. At the Eye and ENT Hospital of Fudan University, every subject had their health assessed.

At Fudan University's Department of Clinical Laboratory, Eye and ENT Hospital, blood tests were conducted. Normally, 4 mL of blood was taken from the cubital veins of fasting volunteers in the morning, held at room temperature for 30 minutes, and then centrifuged at 3,000 rpm for 10 minutes. The levels of serum direct bilirubin (DBIL) and total bilirubin (TBIL) were assessed by Roche Cobas 8000 C702 (Mannheim, Germany). Enzymatic colorimetry was used to determine the levels of the serum enzymes aspartate aminotransferase (AST), alanine transaminase (ALT), and gamma glutamyl transpeptidase (GGT) (Roche Cobas 8000 C702, Mannheim, Germany). To ensure the accuracy of the detection system, a Roche C702 automatic biochemical instrument was used to test indoor quality control every day, and the variation coefficient each month was controlled at 2%–6%.

### 2.3. Diagnostic and Inclusion Criteria

#### 2.3.1. POAG Subjects

In total, 276 participants were recruited, of whom 78 were excluded (secondary glaucoma = 22, congenital glaucoma = 11, other concomitant eye diseases = 10, neovascular glaucoma = 9, systemic diseases = 6, thyroid dysfunction = 8, hepatic diseases = 6, macular degeneration = 5, and cancer = 1), leaving a final sample size of 198 patients.

POAG subjects (1) aged 18 years old or older; (2) have an IOP greater than 21 mmHg; (3) exhibited glaucomatous optic neuropathy, such as high vertical cup-disk ratios(VCDR) > 0.7 or intereye asymmetry > 0.2, with notching, rim thinning, or retinal nerve fiber layer (RNFL) defects; (4) possessed an anterior chamber angle that was considered open and normal in appearance on gonioscopy in both eyes [16]; (5) experienced structurally related visual field impairments (existence of at least three contiguous nonedged test points on the corrected probability plot at *p* < 0.05, with at least one point at p 0.01, omitting points directly above and below the blind spot); and (6) have other eye diseases (in addition to senile cataracts) being ruled out. The study excluded patients with secondary glaucoma, congenital glaucoma, prior intraocular surgery, and ocular diseases that might impair the visual field, such as optic disk abnormalities, optic nerve damage, retinal disease, pathological myopia, and intracranial lesions. Based on a review of systemic diseases, patients who met the following criteria were excluded: (1) below 18 years of age or pregnant; (2) acute infectious or autoimmune diseases or exhibited metabolic syndrome [17]; (3) serious cardiovascular (coronary heart disease, heart failure, and stroke) [18], liver, or kidney diseases; (4) recent eye surgery or trauma; and (5) cancer diagnosis.

#### 2.3.2. Control Subjects

During the research period, those who took part in yearly health examinations were used to find healthy controls. In addition, all healthy subjects underwent various ophthalmic examinations, including refractive status testing, angle mirror examinations, and slit-lamp examinations. Normal subjects were defined as follows: no glaucoma or other eye disease that could affect vision or vision function, no recent eye surgery, no acute infectious disease, no systemic metabolic disease, no autoimmune disease, and no cancer. The final sample consisted of 205 control patients after 27 were eliminated due to the inclusion criteria (cataract = 9, cancer = 4, liver disease = 4, autoimmune disorder = 3, macular degeneration = 2, and other disorders = 5).

### 2.4. Sample Size

The two-tailed assumed sample size was calculated by the formula below, with a type I error rate < 0.05 (*α* = 0.05) and a type II error rate of 0.20 (power: 1–*β* = 80%):

(1) $$\begin{array}{c} n={\left(\left(Z1-\frac{\alpha }{2}\right)+\left(Z1-\beta \right)\right)}^{2}\times \frac{\sigma 1^{2}+\sigma 2^{2}}{\delta^{2}}, \end{array}$$

where *σ* is the standard deviation, *δ* is the expected mean difference, *Z*1 − *α*/2 = 1.96, and *Z*1 − *β* = 0.84.

Based on the above-mentioned assumptions, a sample size of 152 cases of TBIL was required. Therefore, the minimal sample size in this study was 152 cases.

### 2.5. Statistical Analyses

Version 13.0 of the Statistical Package for the Social Sciences was used to conduct the analyses (SPSS Inc., Chicago, IL, USA). The outcomes are displayed as mean standard deviation (SD). To establish normalcy, the Kolmogorov-Smirnov test was employed. To assess the TBIL's capacity for discriminating, ROC curve (receiver operating characteristic) analysis was done. To compare the participant characteristics between the groups, an independent Student's *t*-test and chi-squared test were used. To compare the TBIL levels of the two groups, an ANOVA was used. Using Pearson's correlation analysis, the relationship between cholesterol levels and clinical indicators was assessed. Through the use of multiple linear regression analysis, the connection between TBIL levels and clinical indicators was evaluated. The risk variables for POAG were discovered through the use of logistic regression analysis. The results that had a *p* value lower than 0.05 were deemed statistically significant.

## 3. Results

### 3.1. Characteristics of the Study Patients

For this study, a total of 205 control participants (male = 106 and female = 99) and 198 POAG subjects (male = 117 and female = 81) were included. The mean ages of the control group and POAG were, respectively, 57.50 ± 12.02 years and 59.05 ± 13.21 years. Only one eye was chosen at random if a person had POAG in both of their eyes. The mean ages and genders of the POAG and control groups were nearly identical (*p* = 0.221 and 0.137, respectively). Compared to the control group, serum TBIL levels were significantly increased (*p* < 0.05). In the male subgroup, the serum TBIL and DBIL levels were significantly higher (*p* < 0.05) in the POAG group than in the control group, while there was no significant difference between the serum TBIL and DBIL levels in the female POAG and control groups. There was no statistical difference in the mean levels of AST, ALT, and GGT between the POAG and control subjects (*p* > 0.05). The results of the ROC analysis showed that the discrimination ability of TBIL was limited (*p* > 0.05) (Supplementary Figure 1).  Table 1 shows the results of the analysis of the demographics and serum lipid levels of the POAG and control groups.

### 3.2. Comparing the Characteristics, Bilirubin Levels, and Ocular Parameters in Individuals with POAG, Grouped by Severity

Based on the VCDR, the subjects were divided into three subgroups according to the severity of the disease: 44 mild patients, 69 moderate patients, and 93 severe patients.  Table 2 presents a comparison of ocular and platelet parameters in POAG participants. There was no significant difference in age among the three groups (*p* = 0.915). TBIL and DBIL levels were the highest in the severe POAG group (TBIL: 12.55 ± 5.67 *μ*mol/L; DBIL: 4.55 ± 2.20 *μ*mol/L), followed by the moderate POAG group (TBIL: 11.12 ± 5.03 *μ*mol/L; DBIL: 4.23 ± 1.80 *μ*mol/L) and the mild POAG group (TBIL: 10.34 ± 3.85 *μ*mol/L; DBIL: 3.52 ± 1.23 *μ*mol/L). There were statistically significant differences between mild and severe POAG and between moderate and severe POAG (*p* < 0.05), as shown in Table 2.

### 3.3. Comparison of Serum Bilirubin Levels between Different Severity Subgroups of POAG Based on Gender

The POAG group was divided into male and female subgroups with different severities. The male subgroup included 17 mild patients, 43 moderate patients, and 57 severe patients. The serum TBIL levels were the highest in the severe POAG group (14.47 ± 5.45 *μ*mol/L), followed by the moderate POAG group (12.00 ± 5.55 *μ*mol/L) and the mild POAG group (10.82 ± 4.48 *μ*mol/L). The results of an LSD post hoc test (*p* < 0.05) showed statistically significant differences between mild and severe POAG and moderate and severe POAG (Table 3).

The female subgroup included 27 mild patients, 23 moderate patients, and 31 severe patients. The results of an LSD post hoc test (*p* > 0.05) showed no significant differences in serum TBIL levels between the mild and severe POAG groups and the moderate and severe POAG groups (Table 4).

### 3.4. Analysis of Serum Bilirubin Levels in POAG and Control Subjects Using Logistic Regression

The results of the logistic regression analyses revealed that serum TBIL (odds ratio (OR) = 1.079, 95% confidence interval (CI) = 1.029–1.132) and DBIL (OR = 1.206, 95% CI = 1.060–1.372) levels were positively correlated with POAG after adjusting for age and other demographic parameters (Table 5).

In the male subgroup, the results of the logistic regression analyses revealed that serum TBIL levels (OR = 1.126, 95% CI = 1.009–1.256) were positively correlated with POAG after adjusting for age and other demographic parameters that are independent risk factors for POAG (Table 5).

In the female subgroup, the results of the logistic regression analyses revealed that serum TBIL (OR = 0.969, 95% CI = 0.847–1.109) and DBIL (OR = 1.076, 95% CI = 0.747–1.549) levels were not associated with POAG after adjusting for age and other demographic parameters (Table 5).

### 3.5. Correlation for Associations between Serum Bilirubin and Ocular Parameters in Patients with POAG

Pearson's correlation analysis showed that serum TBIL levels were positively correlated with IOP (*r* = 0.134, *p* < 0.05); VCDR (*r* = 0.142, *p* < 0.05); ACD (*r* = 0.190, *p* < 0.05); and AL (*r* = 0.179, *p* < 0.05). DBIL levels were positively correlated with VCDR (*r* = 0.162, *p* < 0.05) and MD (*r* = 0.166, *p* < 0.05), but negatively correlated with MS (*r* = −0.202, *p* < 0.05) (Table 6).

The results of Pearson's analysis showed that in the male subgroup, serum TBIL levels were positively correlated with VCDR (*r* = 0.218, *p* < 0.05) and negatively correlated with MS (*r* = −0.242, *p* < 0.05), as shown in Table 7. In the female subgroup, there was no correlation between TBIL levels and VCDR or MS, as shown in Table 8.

## 4. Discussion

Few earlier literatures, to our knowledge, have investigated at the connection between blood TBIL levels and POAG. Serum TBIL levels in our study's POAG patients were noticeably greater than those in the control group's participants. The group with severe POAG had the highest TBIL levels, followed by the groups with moderate and mild POAG. In the male subgroup, the greater severity of the disease corresponded to higher levels of TBIL, but there was no such phenomenon in the female subgroup. The findings of the logistic regression study revealed a favorable correlation between TBIL and the seriousness of POAG. The findings also revealed that, in contrast to the female sample, the male subgroup's TBIL levels substantially linked with POAG. The results demonstrated that TBIL levels in serum were significantly correlated with the possibility of POAG and were risk factor for POAG.

Although the pathogenesis of glaucoma is complex and remains unclear, the role of oxidative and antioxidant status disorders has been studied in both patient and animal models. Oxidative stress is caused by the formation of excess reactive oxygen species (ROS) by the human body in a pathological state that exceeds the body's antioxidant capacity. This can lead to cell death and the accumulation of apoptotic residues, as well as the production of autoantibodies and the activation of autoimmune cascades. Previous research has shown that bilirubin, an endogenous antioxidant, is closely involved in the oxidative stress response in the human body [19].

However, bilirubin may be a double-edged sword. On the one hand, elevated bilirubin levels have been linked to oxidative stress and neurotoxicity [20]. The neurotoxicity caused by bilirubin is the result of intricate cellular and molecular processes. It affects specific areas of the central nervous system and changes the blood-brain barrier. Numerous changes in neurons, glial cells, and cellular and mitochondrial membranes might result from high bilirubin levels. Additionally, bilirubin causes abnormalities and can potentially stop the cell cycle by inducing oxidative stress and inflammatory factor cascades. However, molecular biology research has shown that bilirubin is a strong antioxidant [21]. Previous studies have shown that the production of bilirubin is related to the antioxidant defense mechanism and that higher concentrations of bilirubin correspond to lower incidences of oxygen free radical-mediated injury [22, 23]. In addition, related epidemiological studies have shown that bilirubin levels are closely negatively correlated with the occurrence and development of cardiovascular disease [24, 25]. It has been suggested that bilirubin also has a potential cytotoxic effect [26]. Toxicological studies have shown that high concentrations of bilirubin in serum can cause bilirubin to bind with and deposit on various tissues in the body, resulting in jaundice, cerebral palsy, mental disorders, brain damage, and even death [9]. Glaucoma is a type of optic neurodegenerative disease in which the metabolism in the brain is impaired [27]. Glaucoma is characterized by decreased visual acuity and atrophy of the visual papilla. Retinal ganglion cell and axon progression defects are considered the main pathological changes in glaucoma [28, 29]. Therefore, high TBIL levels may increase the toxicity experienced by optic nerve cells in patients with POAG, which is closely related to the occurrence of POAG. This suggests that serum bilirubin is closely related to POAG development.

Furthermore, we examined the relationship between TBIL levels and the severity of POAG, classifying it into the following levels: mild, moderate, and severe. The results showed that serum TBIL levels were the lowest in the mild glaucoma group, followed by the moderate glaucoma group and the severe glaucoma group (*p* = 0.016). High levels of bilirubin can be toxic to the nervous system. Furthermore, the results of Pearson's analysis showed that TBIL levels had a positive correlation with IOP (*p* = 0.012), ACD (*p* = 0.014), and AL (*p* = 0.019); DBIL levels had a positive association with MD (*p* = 0.037) and a negative association with MS (*p* = 0.013). These results indicate that high bilirubin levels may be involved in IOP elevation and bilirubin-induced neurologic dysfunction. In other words, our study found that as the severity of glaucoma gradually increased, the level of TBIL also gradually increased, possibly because the higher the concentration of TBIL, the more serious the toxic damage to the ocular optic nerve cells.

A recent study of 70 POAG patients found significantly lower TBIL levels than in the controls [15], which is contrary to the findings of our study. Potential reasons may be the following: first, differences in sample size (70 vs. 198) may result in inconsistent results; second, our study conducted gender subgroup analyses and found that TBIL levels were significantly correlated with POAG in the male subgroup but not in the female subgroup; mixed male and female analyses may also yield inconsistent conclusions.

In this study, the correlation between TBIL levels and POAG varied between genders. In the male subgroup, TBIL levels were high in patients with POAG, and TBIL levels increased with the severity of POAG in a statistically significant manner. The results of the logistic regression analyses revealed that serum TBIL levels were positively correlated with POAG after adjusting for age and other demographic parameters. However, no statistically significant was observed in the female subgroup. Liu et al. [30] showed gender differences in the correlation between TBIL and fundus arteriosclerosis, where high TBIL levels were associated with fundus arteriosclerosis in men, but no association was found between TBIL and fundus arteriosclerosis in women. Previous studies have shown that the concentration of bilirubin in human serum is correlated with gender and that the average concentration of all kinds of bilirubin in males is higher than in females [31]. These findings, combined with our results, suggest that TBIL levels are associated differently with POAG based on gender.

The following are the limitations of our investigation. Firstly, the fact that this study was cross-sectional case-control restricted our capacity to investigate the precise mechanism behind the correlation between TBIL and POAG as well as to precisely determine the causative connection between TBIL and POAG. Therefore, to further understand the connection between TBIL and POAG, large-scale multicenter prospective studies are required in the future. Second, the results may be less generalizable because the data were only gathered at one eye clinic. As a result, more investigation into the part played by TBIL levels in the etiology of POAG is necessary.

## 5. Conclusion

In this study, we found that patients with POAG had higher TBIL levels, which were significantly positively correlated with POAG severity in male patients, but not in female patients. Thus, TBIL may be related to the pathogenesis and development of POAG, and there is a clear correlation with gender.

## Figures and Tables

**Table 1:** Demographics parameters and serum TBIL levels of the PACG and control groups.

	POAG group (*N* = 198)	Control group (*N* = 205)	*T* value	*p* value
Age (years)	59.05 ± 13.21	57.50 ± 12.02	-1.225	0.221
Gender (male/female)	117/81	106/99	1.491	0.137
VCDR	0.78 ± 0.18			
IOP (mmHg)	20.14 ± 8.69			
ACD (mm)	3.09 ± 2.50			
AL (mm)	24.78 ± 2.27			
CCT (mm)	531.23 ± 40.54			
MD (dB)	16.07 ± 7.94			
MS (dB)	11.61 ± 7.92			
TBIL (*μ*mol/L)	11.58 ± 5.16	10.18 ± 3.38	-3.207	0.001
Male	13.03 ± 5.51	10.77 ± 3.32	-3.665	<0.001
Female	9.48 ± 3.74	9.54 ± 3.35	0.112	0.911
DBIL (*μ*mol/L)	4.21 ± 1.92	3.74 ± 1.24	-2.918	0.004
Male	4.68 ± 2.06	3.99 ± 1.27	-2.958	0.003
Female	3.54 ± 1.48	3.47 ± 1.16	-0.366	0.715
ALT (U/L)	24.00 ± 12.59	22.50 ± 14.19	-1.116	0.264
Male	26.88 ± 13.48	25.61 ± 15.13	-0.657	0.512
Female	19.84 ± 9.86	19.14 ± 12.30	0.421	0.675
AST (U/L)	21.29 ± 6.59	21.09 ± 7.11	-0.299	0.765
Male	21.78 ± 7.11	22.30 ± 7.82	0.522	0.602
Female	20.59 ± 5.74	19.78 ± 6.02	-0.927	0.355
GGT (U/L)	28.41 ± 19.08	29.06 ± 20.98	0.327	0.655
Male	32.05 ± 19.86	35.84 ± 23.15	1.305	0.193
Female	23.15 ± 16.64	21.73 ± 15.37	-0.585	0.559

VCDR: vertical cup/disc ratio; CCT: central corneal thickness; ACD: anterior chamber depth; AL: axial length; MD: mean deviation values for the visual field; MS: mean sensitivity values for the visual field; IOP: intraocular pressure; POAG: primary open-angle glaucoma; TBIL: total bilirubin; DBIL: direct bilirubin; AST: aspartate aminotransferase; ALT: cereal third transaminase; GGT: *γ*-glutamyl transpeptidase. The independent Student *t*-test and chi-squared test were used.

**Table 2:** Comparison of serum bilirubin levels among POAG groups with different severities of disease.

Factors	Mild POAG (*N* = 44)	Moderate POAG (*N* = 69)	Severe POAG (*N* = 93)	*p* value
Age (y)	59.77 ± 14.37	58.94 ± 12.69	58.76 ± 13.13	0.915
Male/female	17/27	43/23	57/31	0.007^ab^
VCDR	0.50 ± 0.10	0.76 ± 0.05	0.93 ± 0.05	<0.001abc
IOP (mmHg)	19.33 ± 7.79	19.52 ± 8.05	21.00 ± 9.55	0.456
ACD (mm)	2.77 ± 0.56	3.34 ± 3.32	3.08 ± 2.42	0.574
AL (mm)	24.26 ± 1.88	24.85 ± 2.00	24.98 ± 2.55	0.258
CCT (mm)	537.78 ± 46.73	531.67 ± 39.27	527.73 ± 38.24	0.438
MD (dB)	8.31 ± 5.47	14.57 ± 7.01	20.66 ± 6.23	<0.001abc
MS (dB)	18.94 ± 5.95	13.69 ± 6.66	6.90 ± 6.24	<0.001abc
TBIL (*μ*mol/L)	10.34 ± 3.85	11.12 ± 5.03	12.55 ± 5.67	0.046bc
DBIL (*μ*mol/L)	3.52 ± 1.23	4.23 ± 1.80	4.55 ± 2.20	0.015bc
ALT (U/L)	25.57 ± 14.78	21.59 ± 11.09	25.02 ± 12.36	0.159
AST (U/L)	22.45 ± 6.88	19.68 ± 4.60	21.92 ± 7.50	0.047
GGT (U/L)	26.75 ± 13.95	26.47 ± 14.64	30.69 ± 23.63	0.322

*χ* ^2^ test and 1-way analysis of variance (ANOVA) were used. Data are expressed as mean ± SD. ^a^*p* < 0.05 for the difference between mild POAG and moderate POAG (1-way ANOVA with the LSD post hoc test). ^b^*p* < 0.05 for the difference between mild POAG and severe POAG (1-way ANOVA with the LSD post hoc test). ^c^*p* < 0.05 for the difference between moderate POAG and severe POAG (1-way ANOVA with the LSD post hoc test). VCDR: vertical cup/disc ratio; CCT: central corneal thickness; ACD: anterior chamber depth; AL: axial length; MD: mean deviation values for the visual field; MS: mean sensitivity values for the visual field; IOP: intraocular pressure; POAG: primary open-angle glaucoma; TBIL: total bilirubin; DBIL: direct bilirubin; AST: aspartate aminotransferase; ALT: cereal third transaminase; GGT: *γ*-glutamyl transpeptidase.

**Table 3:** Comparison of serum bilirubin levels between subgroups of POAG group in male subgroup.

Factors	Mild POAG (*N* = 17)	Moderate POAG (*N* = 43)	Severe POAG (*N* = 57)	*p* value
Age	51.00 ± 12.51	57.77 ± 11.76	57.04 ± 12.99	0.151
VCDR	0.52 ± 0.11	0.76 ± 0.049	0.94 ± 0.048	<0.001
IOP (mm)	20.27 ± 7.25	20.02 ± 8.86	22.13 ± 9.48	0.470
ACD (mm)	2.96 ± 0.40	3.70 ± 4.18	3.36 ± 2.99	0.757
AL (mm)	25.03 ± 2.34	25.17 ± 2.03	25.07 ± 2.29	0.974
CCT (mm)	540.56 ± 42.65	538.83 ± 38.20	532.47 ± 35.55	0.637
MD (dB)	7.48 ± 6.04	14.50 ± 7.25	20.01 ± 6.73	<0.001
MS (dB)	20.40 ± 6.68	13.82 ± 6.68	7.71 ± 6.66	<0.001
TBIL (*μ*mol/L)	10.82 ± 4.48	12.00 ± 5.55	14.47 ± 5.45	0.016
DBIL (*μ*mol/L)	3.88 ± 1.22	4.44 ± 1.98	5.09 ± 2.23	0.067
ALT (U/L)	30.76 ± 16.23	24.56 ± 12.36	27.47 ± 13.33	0.249
AST (U/L)	23.06 ± 5.21	20.00 ± 5.28	22.74 ± 8.50	0.117
GGT (U/L)	31.82 ± 16.24	30.42 ± 15.56	33.35 ± 23.59	0.768

*χ* ^2^ test and 1-way analysis of variance (ANOVA) were used. Data are expressed as mean ± SD. ^a^*p* < 0.05 for the difference between mild POAG and moderate POAG (1-way ANOVA with the LSD post hoc test). ^b^*p* < 0.05 for the difference between mild POAG and severe POAG (1-way ANOVA with the LSD post hoc test). ^c^*p* < 0.05 for the difference between moderate POAG and severe POAG (1-way ANOVA with the LSD post hoc test). VCDR: vertical cup/disc ratio; CCT: central corneal thickness; ACD: anterior chamber depth; AL: axial length; MD: mean deviation values for the visual field; MS: mean sensitivity values for the visual field; IOP: intraocular pressure; POAG: primary open-angle glaucoma; TBIL: total bilirubin; DBIL: direct bilirubin; AST: aspartate aminotransferase; ALT: cereal third transaminase; GGT: *γ*-glutamyl transpeptidase.

**Table 4:** Comparison of serum bilirubin levels between subgroups of POAG group in female subgroup.

Factors	Mild POAG (*N* = 27)	Moderate POAG (*N* = 23)	Severe POAG (*N* = 31)	*p* value
Age	65.30 ± 12.78	61.13 ± 14.29	61.94 ± 13.00	0.489
VCDR	0.49 ± 0.10	0.76 ± 0.05	0.93 ± 0.05	<0.001
IOP (mm)	18.73 ± 8.18	18.60 ± 6.35	18.93 ± 9.47	0.989
ACD (mm)	2.63 ± 0.61	2.76 ± 0.65	2.60 ± 0.51	0.613
AL (mm)	23.81 ± 1.43	24.25 ± 1.86	24.81 ± 3.02	0.292
CCT (mm)	535.92 ± 50.07	519.95 ± 39.01	519.07 ± 41.97	0.323
MD (dB)	8.86 ± 5.15	14.69 ± 6.73	21.82 ± 5.14	<0.001
MS (dB)	17.95 ± 5.35	13.46 ± 6.81	5.49 ± 5.27	<0.001
TBIL (*μ*mol/L)	10.04 ± 3.45	9.48 ± 3.42	9.00 ± 4.23	0.580
DBIL (*μ*mol/L)	3.30 ± 1.20	3.83 ± 1.34	3.55 ± 1.77	0.454
ALT (U/L)	22.30 ± 13.04	16.04 ± 4.71	20.52 ± 8.87	0.072
AST (U/L)	22.07 ± 7.82	19.09 ± 2.92	20.42 ± 4.99	0.183
GGT (U/L)	23.56 ± 11.50	19.09 ± 9.15	25.81 ± 23.29	0.341

*χ* ^2^ test and 1-way analysis of variance (ANOVA) were used. Data are expressed as mean ± SD. ^a^*p* < 0.05 for the difference between mild POAG and moderate POAG (1-way ANOVA with the LSD post hoc test). ^b^*p* < 0.05 for the difference between mild POAG and severe POAG (1-way ANOVA with the LSD post hoc test). ^c^*p* < 0.05 for the difference between moderate POAG and severe POAG (1-way ANOVA with the LSD post hoc test). VCDR: vertical cup/disc ratio; CCT: central corneal thickness; ACD: anterior chamber depth; AL: axial length; MD: mean deviation values for the visual field; MS: mean sensitivity values for the visual field; IOP: intraocular pressure; POAG: primary open-angle glaucoma; TBIL: total bilirubin; DBIL: direct bilirubin; AST: aspartate aminotransferase; ALT, cereal third transaminase; GGT: *γ*-glutamyl transpeptidase.

**Table 5:** Logistic regression analysis of the association between serum bilirubin levels and POAG.

	*B*	*p*	OR (95% CI)
All			
TBIL	0.057	0.002	1.079 (1.029-1.132)
DBIL	0.045	0.004	1.206 (1.060-1.372)
Male			
TBIL	0.119	0.033	1.126 (1.009-1.256)
DBIL	-0.004	0.980	0.996 (0.746-1.331)
Female			
TBIL	-0.031	0.650	0.969 (0.847-1.109)
DBIL	0.073	0.695	1.076 (0.747-1.549)

**Table 6:** Association between serum TBIL and DBIL with ocular parameters.

		IOP (mmHg)	VCDR	CCT (*μ*m)	ACD (mm)	AL (mm)	MD (dB)	MS (dB)
TBIL	*r*	0.134^∗^	0.142^∗^	0.090	0.190^∗^	0.179^∗^	0.093	-0.118
	*p*	0.012	0.046	0.230	0.014	0.019	0.245	0.147
DBIL	*r*	0.101	0.162^∗^	-0.038	0.098	0.066	0.166^∗^	-0.202
	*p*	0.059	0.023	0.615	0.209	0.387	0.037	0.013

VCDR: vertical cup/disc ratio; CCT: central corneal thickness; ACD: anterior chamber depth; AL: axial length; MD: mean deviation values for the visual field; MS: mean sensitivity values for the visual field; IOP: intraocular pressure; POAG: primary open-angle glaucoma; TBIL: total bilirubin; DBIL: direct bilirubin.

**Table 7:** Association between serum TBIL and DBIL with ocular parameters in male subgroup.

		IOP (mmHg)	VCDR	CCT (*μ*m)	ACD (mm)	AL (mm)	MD (dB)	MS (dB)
TBIL	*r*	-0.017	0.218^∗^	-0.040	0.141	0.178	0.200	-0.242^∗^
	*p*	0.857	0.018	0.688	0.172	0.074	0.054	0.023
DBIL	*r*	-0.112	0.163	-0.103	0.060	0.022	0.197	-0.256^∗^
	*p*	0.230	0.079	0.294	0.564	0.827	0.057	0.016

VCDR: vertical cup/disc ratio; CCT: central corneal thickness; ACD: anterior chamber depth; AL: axial length; MD: mean deviation values for the visual field; MS: mean sensitivity values for the visual field; IOP: intraocular pressure; POAG: primary open-angle glaucoma; TBIL: total bilirubin; DBIL: direct bilirubin.

**Table 8:** Association between serum TBIL and DBIL with ocular parameters in female subgroup.

		IOP (mmHg)	VCDR	CCT (*μ*m)	ACD (mm)	AL (mm)	MD (dB)	MS (dB)
TBIL	*r*	0.006	-1.30	0.099	0.131	0.000	-0.121	0.043
	*p*	0.955	0.247	0.397	0.285	0.998	0.342	0.735
DBIL	*r*	-0.052	0.043	-0.125	0.004	-0.043	0.104	-0.149
	*p*	0.648	0.703	0.287	0.972	0.719	0.412	0.244

VCDR: vertical cup/disc ratio; CCT: central corneal thickness; ACD: anterior chamber depth; AL: axial length; MD: mean deviation values for the visual field; MS: mean sensitivity values for the visual field; IOP: intraocular pressure; POAG: primary open-angle glaucoma; TBIL: total bilirubin; DBIL: direct bilirubin.

## Data Availability

The original contributions presented in the study are included in the article, and further inquiries can be directed to the corresponding author.

## Supplementary Materials

Supplementary Materials Figure S1: ROC analysis to diagnose POAG.
