# Supplementary figures and images for: Association between Serum Total Bilirubin Level and Patients with Primary Open-Angle Glaucoma in China: A Cross-Sectional, Case-Control Study
Source: Oxid Med Cell Longev. 2023 Jan 19;2023:8206298. doi: 10.1155/2023/8206298 (PMC9884161; doi:10.1155/2023/8206298)

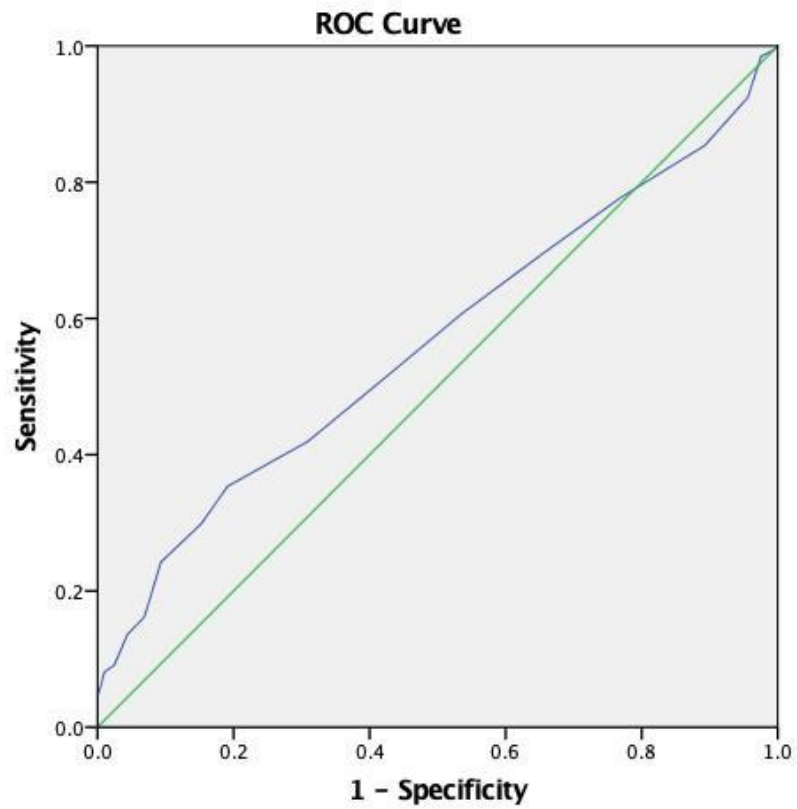

Diagonal segments are produced by ties.

Figure S1. ROC analysis to diagnose POAG.  
AUC=0.565,  $p=0.055$

Supplement: Supplementary Materials — Figure S1: ROC analysis to diagnose POAG. [file 8206298.f1.pdf]
